# Giant cell tumour in the diaphysis of radius – a report

**DOI:** 10.1186/1757-1626-1-106

**Published:** 2008-08-18

**Authors:** Sandeep Shrivastava, Shishir P Nawghare, Yogesh Kolwadkar, Pradeep Singh

**Affiliations:** 1 Department of Orthopaedics and Trauma, DMIMS, Wardha, India; 2 26, MizzyRoad, Rochdale, OL12 6HW, UK

## Abstract

**Background:**

We present a case of a 35 yrs old female who presented with swelling over her forearm. This is a rare case of a giant cell tumour in a nonepiphyseal region.

**Methods:**

Case report and presentation of clinical, radiological and histological data on single case of giant cell tumour of diaphysis of radius.

**Results:**

Although age, clinical features and radiological features are helpful, it is still the histology that helps to clinch the diagnosis.

**Conclusion:**

A thorough literature search and an exhaustive online search using various search engines revealed seven reported cases of giant cell tumours in the diaphysis of long bones. We reiterate the fact that irrespective of the location, a giant cell tumour should be diagnosed based on its histology.

## Introduction

Giant cell tumour is a benign but locally aggressive tumour. The classification and definition of giant cell lesions was first proposed by Jaffe and Lichtenstein[1]. 20% of all benign bone tumours and 5% of all tumours are giant cell tumours. It is more common in young adults between 20 and 40 years of age [2-4]. It is more common in females with the rate of growth enhanced in pregnancy[5].

Appearance before epiphyseal plate closure is rare[2,6,7]. It occurs commonly in the distal femur, the proximal tibia, the distal radius and the sacrum[2-4]. Giant cell tumours (GCT) usually prefers the epiphyses of long bones. The involvement of the metaphysis or diaphysis without epiphyseal extension is quite uncommon[1]. Often the tumour extends to the articular subchondral bone, However it seldom crosses the joint or its capsule. If the GCT appears prior to epiphyseal, it is likely to be found in the metaphysis[8,9]. A diaphyseal GCT is almost unheard of A literature search brought forth very few reported cases[10-14]. This is perhaps the eighth case of diaphyseal GCT reported in the literature. It recurs from time to time and rates between 25–50% have been reported[2,6,15]. In very rare cases, a malignant change may occur[14,16,17]. Taking this into account, it is essential that a correct diagnosis of GCT should be made. It is essential that we are aware of the rare existence of giant-cell tumours in areas other than the epiphysis. We may miss a few if we are not [10].

## Case Report

**Figure 1:**
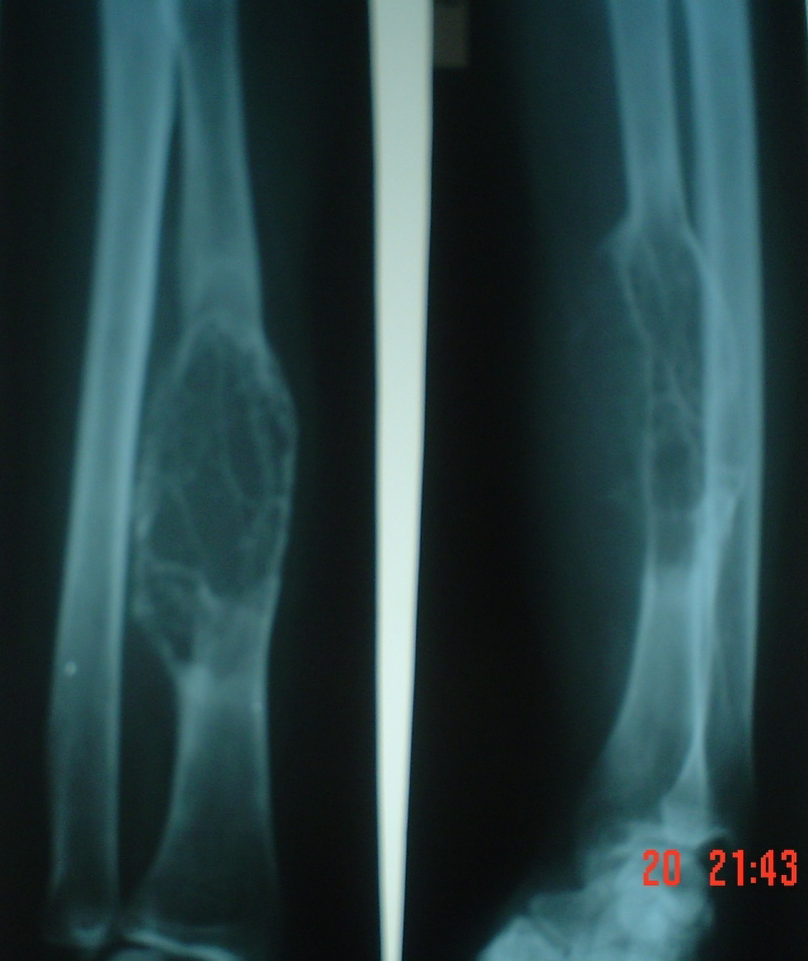
**Radiograph of diaphyseal giant cell tumour**.

A 35 years old Asian school teacher was admitted in our hospital with a complaint of swelling over her left forearm. The swelling had increased gradually over the preceding year. She also complained of occasional pain over the inside of her forearm. She did not sustain any kind of trauma or suffer from any fever in her last few months. Examination revealed a diffuse fusiform swelling over the middle third and outer aspect of her left forearm. The overlying skin was tense. No signs of inflammation were visible. On palpation, there was tenderness over the swelling, especially over the lateral aspect. The swelling was soft in consistency with a feeling of 'egg shell crackling'. Movement at all the joints was full in range and was painless. There was no neurovascular deficit. The calcium, phosphorus and parathyroid levels in the serum were within normal limits. A radiograph of the forearm showed an expansile lesion of 10 cms × 5 cms over the middle third of the radius (Fig 1). The lesion was lytic and ballooned. The cortical margins were thinned out and breached. The chest radiograph was normal. A fine needle aspiration biopsy was done.

**Figure 2:**
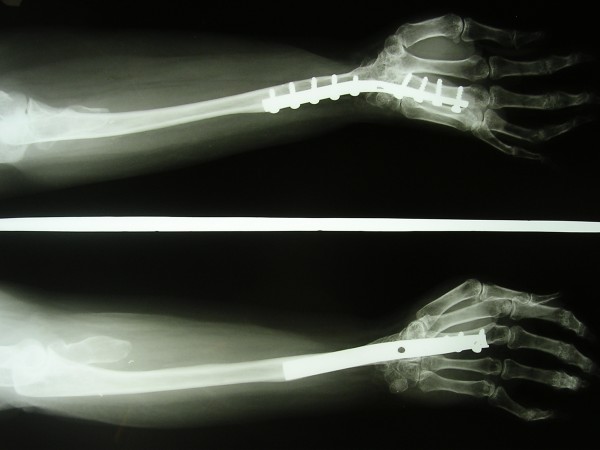
Radiograph-two years postoperative.

To our surprise, it was reported as a giant cell tumour It was decided to excise the tumour. At surgery, the tumour was reddish brown, ovoid in shape and soft in consistency. Frozen section was done to know the extent. It extended from the metaphyseal-diaphyseal junction area of the distal radius to the proximal fourth. It was removed cleanly. The ulna was centralised over the third metacarpal and the wrist was arthrodesed with a dynamic compression plate (Fig 2).

Histology revealed large number of scattered giant cells with centrally placed nuclei. The tumor was composed of plump spindle shaped cells. It was also reported as a giant cell tumour. The tumour did not recur two years after the surgery and the patient is in good health.

## Discussion

Most GCTs are present in the epiphyseal or epimetaphyseal end of the long bones.

If the epiphysis is not involved, a diagnosis of GCT is dubious. The radiographic findings are helpful but cannot clinch the diagnosis. Histological examination is still the gold standard for diagnosis.

To distinguish metaphyseal and diaphyseal GCT from other lesions is a challenge for the pathologist. Several lesions like aneurysmal bone cyst, giant cell-rich osteosarcoma, fibrous cortical defect, solitary (unicameral) bone cyst, and giant cell lesion of hyperparathyroidism (brown tumour) are more common in these sites than are true GCT. This extensive range of lesions with the exception of giant cell lesion of hyperparathyroidism, usually appears in the first two decades of life.

Aneurysmal bone cyst is clinically benign It commonly appears in the metaphysis. It contains prominent blood-filled spaces. The more solid zones within them exhibit fibrogenesis and osteoid trabeculae[12]. The stroma between the spaces contains hemosiderin laden macrophages, chronic inflammatory cells and broad seams of reactive osteoid[5]. Multinucleated giant cells are often conspicuous. Mitoses may be numerous, but anaplasia is absent.

Osteosarcoma is a lesion in the metaphysis and contains numerous benign giant cells. The stroma reveals cells with ananplasia and irregular size and shape. Also presence of cartilage is not uncommon[12].

Fibrous cortical defect is a benign lesion which regresses spontaneously. Radiology shows a characteristic eccentric zone of rarefaction with well-defined scalloped margins[12]. The microscopic picture reveals a mixture of collagen and fibroblasts with irregular cluster of histiocytes filled with lipid and hemosiderin. Multinucleated giant cells may be found[5].

A simple bone cyst generally touches the epiphyseal growth plate. It is benign lesion and shows radiolucence with fine trabeculation. It contains fibrous tissue with few giant cells[12].

A giant cell lesion of hyperparathyroidism appears in the metaphysis. Many giant cells scattered in a fibrogenic stroma may be present. A serum hypercalcemia and hypophosphatemia is characteristic[12].

Giant-cell reparative granulomas demonstrate an appearance which suggests previous injury and inflammation with subsequent fibrosis. These lesions characteristically have an appearance which suggests previous injury and inflammation with subsequent fibrosis. Giant cells are found in the vicinity of old areas of hemorrhage, though not dispersed throughout the lesion. Mandible is a common site for these lesions[10].

The case we have reported did not contain areas of hemorrhage, hemosiderin pigment, osteoid, bone, or significant amounts of collagen. The lesion also did not have the fibrotic, scarred appearance of a fibrous cortical defect, reparative granuloma, or brown tumor of hyperthyroidism. The patient had no detectable parathyroid dysfunction. The clinical and histological features of other osseous lesions which may contain giant cells were not present.

The patient's age, the location of the lesion, its roentgenographic appearance, and the gross and microscopic appearances are crucial to unravel the mystery of an osseus lesion. However, the final diagnosis depends on the tumour's histological appearance only[10]. As Jaffe has mentioned 'A bone lesion may be uncharacteristic in all other respects, but if it exhibits the cytological pattern of a giant cell tumour, it should be recognised as a GCT' [18].

## Competing interests

The authors declare that they have no competing interests.

## Authors' contributions

SS drafted the manuscript and operated on the patient. SPN, YK and PS drafted the manuscript, performed a literature search and participated in the management. All the authors read and approved the final manuscript.

## Consent

Written informed consent was obtained from the patient for publication of this case report and accompanying images.
